# Paralysis from Gun Shot Wound

**Published:** 1861-11

**Authors:** 


### ART. III.—*Paralysis from Gun Shot Wound.*

A. B. from Nebraska, admitted to Surgical Ward, Buffalo General Hospital, service of Dr. Miner, June 20, 1861. Received, while in Nebraska, six months previous to admission, a gun shot wound in the left lumbar region, near the spine, the direction the ball passed or its present location not ascertained. The injury produced immediate paralysis of the body below this point ; urine and feces passed involuntarily ; complete loss of voluntary motion in extremities, but not total loss of sensation. While in this condition was exposed to cold; circulation being feeble, and sensation greatly diminished, the feet were so frozen that the toes sloughed from both feet, and the bones of the toes were removed by the patient, but the metatarsal bones were remaining, and carious. July 20th, operation was made for removal of diseased bones ; the wound healed kindly but slowly ; the region of the tibia upon the other leg not operated upon has been the seat of most violent and protracted pain, the local or general causes of which are not very apparent, and all measures for relief have thus far signally failed ; the paralysis which was at first complete, has gradually subsided, until control is now gained over the rectum and bladder ; tonics, nutritious diet, and exercise of the legs as much as possible, was advised, but, alas ! exercise could not be obtained; patient has greatly improved in general health and strength.

There are some interesting features in this case since the paralysis remains; the foreign body has never been removed and yet there is some improvement in the control of the bladder and rectum. If the exact location of the ball could be ascertained, an operation for its removal might be proper, but unfortunately at the time of injury no surgical attendance could be obtained, and consequently no exploration for the foreign body was made—the case was left to the resources of nature. The paralysis indicates that the ball injured or pressed upon the spinal cord, and that at a point above the second lumbar vertebra since, if in the leash of nerves forming the canda equina, so perfect paraplegia could hardly be expected ; that any improvement in nervous power should have taken place, is somewhat remarkable, and leads us to inquire what could probably have been the nature of the injury to the nervous structure. If from pressure how has it been removed ? if from separation, would it unite so as to form nervous communication ? When nerves are divided by a clean cut there can be no doubt they generally unite so as at length to be restored to almost or quite natural function, the period for this restoration is sometimes very long, and the gradual improvement observed is often attributed to the means adopted for relief the natural course of nature in these cases being forgotten. The difficulties in the way of the regenerative process are greatly enhanced by any loss of substance, contusion or laceration of the ends of the divided nerve.

